# Profiling of Protein-Coding Missense Mutations in Mendelian Rare Diseases: Clues from Structural Bioinformatics

**DOI:** 10.3390/ijms26094072

**Published:** 2025-04-25

**Authors:** Anna Visibelli, Rebecca Finetti, Piero Niccolai, Alfonso Trezza, Ottavia Spiga, Annalisa Santucci, Neri Niccolai

**Affiliations:** 1 Department of Biotechnology, Chemistry and Pharmacy, University of Siena, 53100 Siena, Italy; anna.visibelli2@unisi.it (A.V.); rebecca.finetti2@unisi.it (R.F.); alfonso.trezza2@unisi.it (A.T.); ottavia.spiga@unisi.it (O.S.); neri.niccolai@gmail.com (N.N.); 2 Le Ricerche del BarLume Free Association, Ville di Corsano, Monteroni d’Arbia, 53014 Siena, Italy; pieroniccolai996@gmail.com; 3 Industry 4.0 Competence Center ARTES 4.0 Viale Rinaldo Piaggio, 56025 Pontedera, Italy

**Keywords:** rare diseases, missense mutations, protein structure, molecular dynamics, structural bioinformatics

## Abstract

The growing availability of protein structural data from experimental methods and accurate predictive models provides the opportunity to investigate the molecular origins of rare diseases (RDs) reviewed in the Orpha.net database. In this study, we analyzed the topology of 5728 missense mutation sites involved in Mendelian RDs (MRDs), forming the basis of our structural bioinformatics investigation. Each mutation site was characterized by side-chain position within the overall 3D protein structure and side-chain orientation. Atom depth quantitation, achieved by using SADIC v2.0, allowed the classification of all the mutation sites listed in our database. Particular attention was given to mutations where smaller amino acids replaced bulky, outward-oriented residues in the outer structural layers. Our findings reveal that structural features that could lead to the formation of void spaces in the outer protein region are very frequent. Notably, we identified 722 cases where MRD-associated mutations could generate new surface pockets with the potential to accommodate pharmaceutical ligands. Molecular dynamics (MD) simulations further supported the prevalence of cryptic pocket formation in a subset of drug-binding protein candidates, underscoring their potential for structure-based drug discovery in RDs.

## 1. Introduction

Rare diseases (RDs) are defined by the World Health Organization (WHO) as conditions affecting fewer than 65 individuals per 100,000 people [1]. Despite their rarity, these disorders collectively represent a significant global health burden, with more than 5000 distinct RDs identified according to WHO estimates [2]. This low prevalence often leads to limited investment from the pharmaceutical industry, as the development of treatments for RDs is typically not considered economically viable [3]. As a result, much of the progress in RD research is driven by academic institutions, often under pressure from advocacy groups and non-profit organizations supporting RD patients [4] (see list in [5]). The epidemiological landscape of RDs presents challenges for healthcare systems globally. The lack of comprehensive data for the majority of RDs makes it difficult to accurately assess their true impact across different populations and geographic regions [6]. This knowledge gap, combined with limited expertise and insufficient treatment options, has historically hindered efforts to address these conditions effectively. However, in recent years, RDs have gained increasing recognition as a critical public health issue, leading to international networks to raise awareness and drive research advancements in the field [7,8]. In this context, the Orpha.net database provides a continuously updated and expanding repository of comprehensive knowledge on RDs, aiming to improve healthcare standards and support the delivery of specialized services tailored to the needs of the RD community [9]. Approximately 70% of RDs have a genetic origin [10], with the majority classified as Mendelian disorders [11]. Despite their monogenic nature, over half of the identified Mendelian RDs (MRDs) still lack a clear genetic explanation [12]. Even when the causative gene mutation has been identified, patients often exhibit diverse phenotypes, complicating diagnosis and treatment approaches. This variability reflects the complex, non-linear relationship between genotypes and phenotypes, which remains a fundamental challenge in RD research. Given the genetic basis of MRDs, genome editing technologies have emerged as promising strategies to limit the complications associated with MRDs [13]. However, such advanced therapies may remain inaccessible to patients in low-income regions or disadvantaged social conditions, highlighting the need for more widely accessible treatment options like orally available drugs [14].

Missense variants, which result in alterations to a protein’s amino acid sequence, represent a significant proportion of the genetic mutations associated with MRDs. These variants can be classified as either pathogenic or benign, with pathogenic variants disrupting protein function, whereas benign variants typically have limited impact. Despite their prevalence, only about 2% of all missense variants have been definitively classified, presenting a critical knowledge gap that impedes clinical diagnosis and treatment of RDs [15]. Notably, missense mutations can generate new surface cavities that disrupt normal protein physiological function [16], and, in some cases, these defects can be corrected with rationally designed drugs [17], suggesting that damaged protein functions can sometimes, in principle, be rescued [18]. Structural rescue occurs when a small molecule binds to a destabilized region of a mutated protein, compensating for the mutation-induced instability and restoring function. This process often involves stabilizing the native fold of the protein, preventing misfolding, and allowing it to regain its biological activity. Structural rescue is particularly useful in cases where mutations create druggable cavities or disrupt key functional regions, making them amenable to targeted pharmacological intervention [19,20]. The identification and design of small molecules that interact with these sites let researchers develop therapies that mitigate the pathogenic effects of missense mutations, ultimately restoring protein function. This principle has been successfully applied to restore the tumor-suppressing activity of *p53* missense variants [17], highlighting a potential avenue for therapeutic intervention in genetic diseases. Structure-based approaches thus provide a viable alternative to gene therapies, offering a pharmacological route to restoring protein function and expanding treatment possibilities for patients. Similarly, this approach has been used in cystic fibrosis, where small molecules stabilize the defective CFTR protein and enhance its function [21], as well as in Fabry disease, where pharmacological chaperones like migalastat help restore enzymatic activity by stabilizing the mutant enzyme [22]. Structure-based approaches thus provide a viable alternative to gene therapies, offering a pharmacological route to restoring protein function and expanding treatment possibilities for patients.

**Figure 1:**
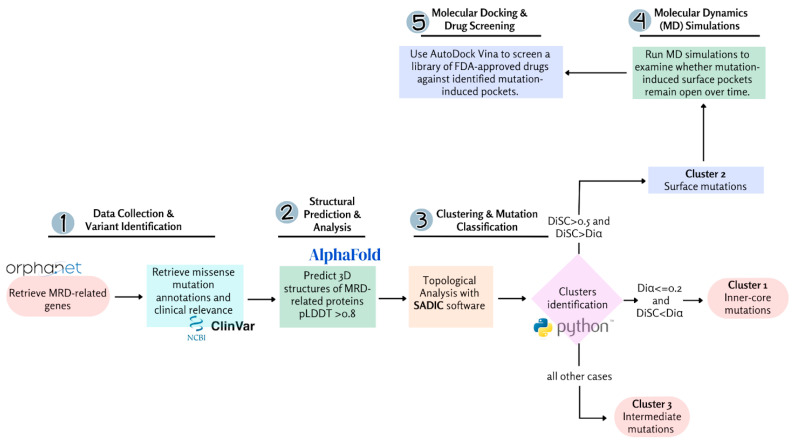
The workflow consists of five key steps: (**1**) Data collection and variant identification, where Mendelian Rare Disease (MRD)-related genes and missense mutation annotations are retrieved from Orpha.net and ClinVar; (**2**) structural prediction and Analysis, where AlphaFold predicts 3D structures of MRD-related proteins; (**3**) clustering and mutation classification, using topological analysis with SADIC software to classify mutations into inner-core, intermediate, and surface clusters; (**4**) molecular dynamics (MD) simulations, to assess mutation-induced surface pocket stability; and (**5**) molecular docking and drug screening, where AutoDock Vina screens FDA-approved drugs for potential binding to these pockets.

Recent advances in artificial intelligence (AI) and computational biology have expanded our understanding of protein structures [23]. The AlphaFold database now provides millions of accurately calculated protein structure models, covering a substantial portion of UniProtKB content [24]. This structural information, combined with bioinformatics approaches, enables detailed topological analyses of mutation sites in MRD-related missense variants [25,26]. Additionally, AI-driven tools such as AlphaMissense offer novel capabilities to estimate the pathogenicity of all known missense genomic variants [27,28]. In this study, we employ a novel structural bioinformatics approach to investigate whether a similar structure-based drug design approach can be applied to MRDs. Specifically, we aim to identify missense mutations reviewed by Orpha.net that generate druggable surface cavities, potentially paving the way for small-molecule therapeutics to rescue defective protein function. The workflow of this study is summarized in Figure 1.

## 2. Results

As of 17 September 2024, the Orpha.net database classified 3889 conditions as RD and identified 1290 genes involved in MRD through missense mutations. The ClinVar database integrated all of the diseases and associated genes from Orpha.net, enhancing the dataset with information on the molecular consequences of reported variants.

For the subsequent structural analysis, it was essential to obtain structural information for all MRD-related proteins, including those lacking experimentally determined structures. To overcome this limitation, AlphaFold was used to predict the three-dimensional structures of all proteins in the dataset. Only predictions with an average protein pLDDT score > 0.8 and a pLDDT score > 0.8 at the specific residue position were considered, resulting in a final dataset of 585 proteins for analysis.

Given that MRD-related proteins often carry multiple mutations, we examined the topology of 5728 missense mutation sites, as detailed in Table S1.

A key focus of this analysis was to identify mutations that could create novel cavities on the mutant protein surface. These structural changes are particularly relevant for developing potential pharmacological approaches for MRDs listed by Orpha.net. Moreover, the impact of each mutation depends on whether the mutated side chain is oriented toward the core or the surface of the protein, as well as its atom depth, leading to different structural and functional consequences.

**Figure 2:**
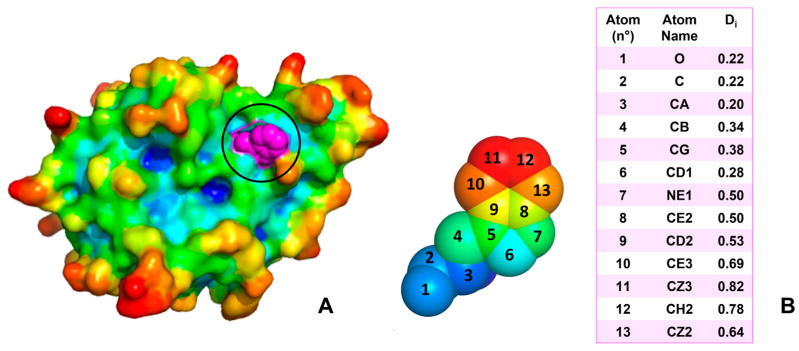
Atom depth analysis of amino acid orientation. (**A**) Crystal structure of human dihydropteridine reductase (PDB ID: 1HDR) with Trp107 highlighted in magenta (highlighted with black circle). Replacement of this residue with Gly is associated with an MRD. The Trp107 side chain, characterized by a high D_iSC_/D_iα_ ratio, indicates an outward orientation. (**B**) Detailed view of Trp107 with atoms numbered according to PDB nomenclature and colored based on their SADIC-calculated depth indexes (D_i_), corresponding to values presented in the table.

To quantify these aspects, we assessed the overall protein structure and solvent accessibility of each mutated residue. Side chain positions and directions of residues involved in MRD-inducing mutations were determined by calculating tridimensional atom depths with SADIC software. The directionality of each mutated side chain was characterized by comparing the depth index of the C_α_ atom (D_iα_) with the average depth index of the side chain atoms (D_iSC_). If D_iα_ was lower than D_iSC_, the side chain was considered to be oriented toward the protein surface, as shown in Figure 2.

Based on this criterion, we performed a preliminary topological classification of MRD mutations (see Table S1). Amino acids were classified into three clusters, as shown in Table 1. Residues with D_iα_ values below 0.2 with D_iSC_ < D_iα_ were assigned to the inner layer, those with D_iSC_ values above 0.5 and D_iSC_ > D_iα_ were placed in the outer layer, while all the other cases were classified as intermediate layers.

For the outer layer (D_iSC_ > 0.5 and D_iSC_ > D_iα_), we focused on large and charged residues, while for the inner layer (D_iSC_ < D_iα_ and D_iα_ ≤ 0.2), we selected smaller or hydrophobic residues. Applying these thresholds allowed us to identify structural constraints under which MRD-inducing mutations may be responsible for the creation of new surface cavities.

**Figure 3:**
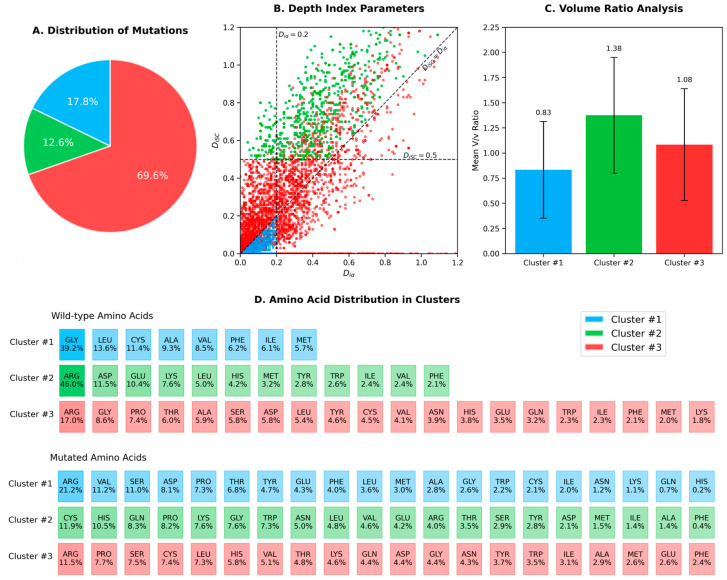
Analysis of protein mutations divided into three clusters, each represented by distinct colors (blue for Cluster #1, green for Cluster #2, and red for Cluster #3). (**A**) Distribution of mutations among the three clusters. (**B**) Scatter plot of depth index parameters, with points colored according to their cluster assignment. (**C**) Volume ratio analysis, showing the mean *v*/*v* ratio for each cluster with error bars. (**D**) Amino acid distribution in each cluster, divided into two sections: wild-type amino acids (**top**) and mutated amino acids (**bottom**). Each colored cell represents the frequency of specific amino acids within each cluster, with percentages indicated.

To better understand the characteristics of the three clusters, we performed an integrated analysis of their distribution, spatial organization, and amino acid composition. Figure 3 displays a comprehensive visualization of our findings.

## 3. Discussion

Our structural bioinformatics analysis of missense mutation sites involved in MRDs has revealed distinct topological patterns that provide insights into their molecular mechanisms and potential therapeutic approaches.

Cluster #1 (inner layer) contained 1018 mutations (17.8% of the total), characterized by inward-pointing side chains. These mutations predominantly involved hydrophobic or small amino acids located in the protein core. Notably, mutations in this cluster frequently disrupt the hydrophobic interactions essential for proper protein folding, potentially leading to protein destabilization and loss of function. Establishing the absence of a specific protein in the human proteome is a complex task [29], and the current structural bioinformatics approach can significantly contribute to identifying this issue by directly analyzing the position and side chain orientation of the mutated amino acids. In these cases, MRD therapeutic solutions are likely achievable only through genome editing techniques, mRNA-based protein replacement therapies [30], or by engineering microorganisms or cells to produce the missing protein for external administration [31].

Cluster #3 (intermediate layer) comprised mutations that do not fall into the specific inward or outward orientation categories defined for Cluster #1 and Cluster #2. Despite representing the largest set of mutations, with 3988 occurrences (69.6% of the total), this group falls outside the scope of the present investigation.

**Figure 4:**
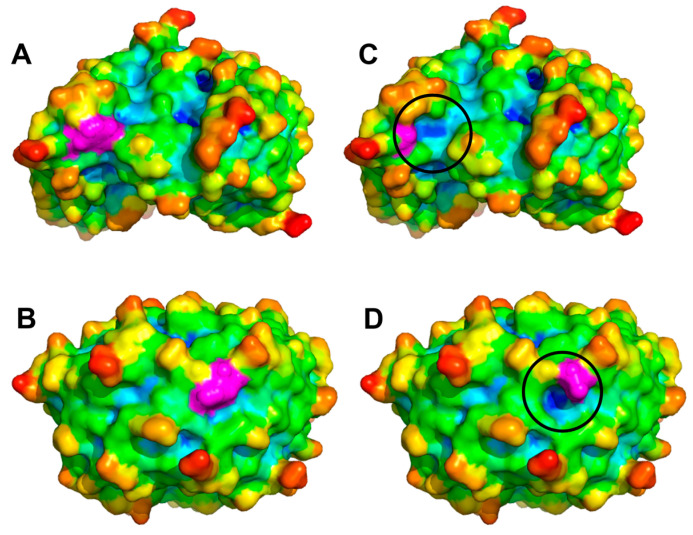
Crystal structures of wild-type and mutated proteins involved in MRD. (**A**,**B**) Surface representations of PDB structures 3S5N and 7MT1, respectively, colored according to atom depth index, with residues involved in MRD-causing mutations highlighted in magenta. (**C**,**D**) Visualization of the void spaces (highlighted with black circles) created by Trp142Gly and Phe142Ser replacements in 3S5N and 7MT1, respectively.

Cluster #2 (outer layer) included 722 mutations (12.6% of the total), featuring outward-pointing side chains. This cluster primarily contained bulky or charged amino acids located on the protein surface. The outward orientation of the mutated side chain can facilitate the formation of mutation-induced cavities, particularly when the substitutions involve significant changes in the side chain steric hindrance or electric charge network. A detailed analysis of amino acid substitutions within this cluster revealed that 381 mutations (52.8% of Cluster #2) involved the replacement of larger residues with smaller ones (*V*/*v* > 1.3). Representative examples of Cluster #2 mutations leading to such structural changes are shown in Figure 4.

Despite the therapeutic potential of Cluster #2 mutations, it is important to consider that local conformational rearrangements may compensate for the structural void, preventing the formation of stable binding pockets. This dynamic nature of protein surfaces poses a challenge in identifying cavities suitable for drug targeting. To overcome this, we employed MD simulations to verify whether MRD-associated mutations in Cluster #2 consistently lead to the formation of new ligand-binding sites. Table 2 lists the proteins selected for MD simulations, each carrying MRD mutations that may induce the formation of new binding pockets. For these proteins, the corresponding three-dimensional structures were retrieved from the PDB to facilitate structural analysis. These proteins were chosen not only for their mutational profiles but also because their three-dimensional structures were available in the PDB, enabling a more precise structural analysis. These mutations exhibit favorable side chain orientations of the replaced residue, quantified by D_iSC_/D_iα_ ratios, and are associated with a suitable volume reduction between the wild-type and mutated amino acid, underscoring their potential structural impact.

**Figure 5:**
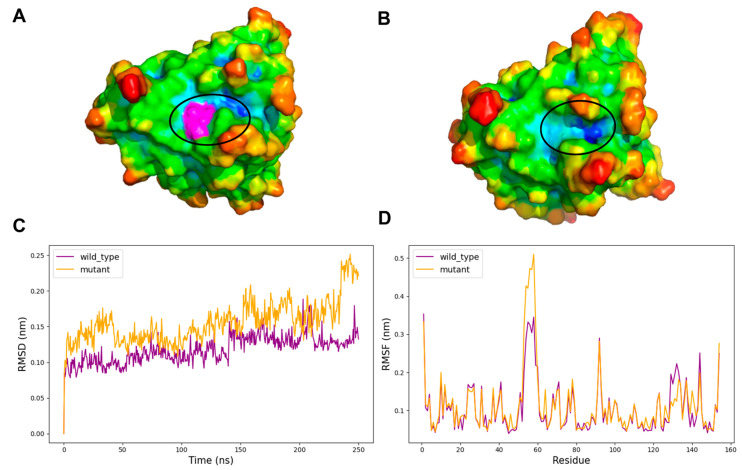
Structural and dynamic analysis of *SOD1* in wild-type and Val149Gly mutant forms. (**A**) Surface representation of the wild-type *SOD1* (PDB ID: 8GSQ) colored according to atom depth indexes obtained with SADIC v2.0, with Val149 highlighted in magenta and circled to indicate its position. (**B**) Surface representation of the Val149Gly mutant model showing the altered surface topography at the mutation site (circled). (**C**) root mean square deviation (RMSD) plot from 250 ns molecular dynamics simulations comparing backbone stability of wild-type (purple) and mutant (orange) *SOD1* structures. (**D**) root mean square fluctuation (RMSF) analysis per residue, comparing local flexibility between wild-type (purple) and mutant (orange) structures.

Thus, we conducted 250 ns MD simulations in explicit solvent for both wild-type and MRD-mutated proteins, generated using the DUET tool. Full MD simulation metrics are provided in the Supporting Information. Structural analysis of the MD trajectories revealed that, among the cases examined, the Val149Gly mutation in *SOD1* led to a detectable change in the surface shape, with a pocket disclosure that could hold a pharmacologically active compound (see Figure 5A,B).

The root mean square deviation (RMSD) analysis (Figure 5C) reveals distinct conformational behaviors between the wild-type and Val149Gly mutant *SOD1* structures throughout the simulation. While the wild-type structure maintains relatively stable RMSD values of around 0.10–0.13 nm, the mutant exhibits increased backbone flexibility, reaching up to 0.25 nm by the end of the 250 ns trajectory. This difference suggests that the Val149Gly mutation induces broader conformational changes beyond the immediate mutation site, potentially affecting the protein’s overall stability. The root mean square fluctuation (RMSF) plot (Figure 5D) provides residue-specific information about local flexibility and further illuminates the impact of the Val149Gly mutation. Interestingly, residues comprising the mutant-formed pocket show complex patterns of altered dynamics. While residues 147–151, which include the mutation site itself, display modest changes in flexibility, more pronounced effects are observed in residues 52–54, where the mutant exhibits higher RMSF values. Therefore, the mutation could trigger allosteric effects that propagate to distal regions of the protein structure. Although the increased flexibility in these regions likely contributes to the pocket formation observed in the mutant structure, the pocket residues maintain sufficient structural integrity to form a defined cavity capable of potentially accommodating pharmacological compounds.

**Figure 6:**
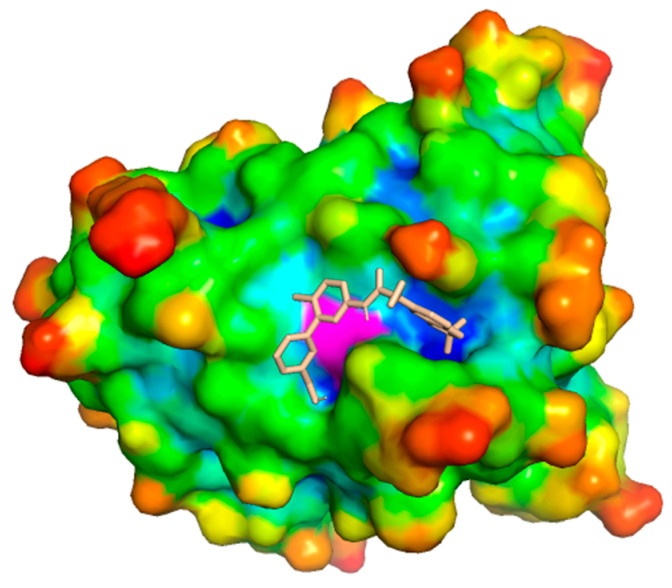
Molecular docking of the small molecule (PubChem ID: 16678941) into the surface of the mutated *SOD1* protein (PDB: 8GSQ, Val149Gly mutation). The receptor is displayed as a molecular surface colored according to atom depth indexes obtained by using SADIC v2.0, apart from the magenta Gly149, with the docked ligand shown in stick representation. The mutation at position 149 (Val → Gly) may induce structural rearrangements that facilitate ligand binding, potentially altering protein function.

To further assess the potential applicability of this newly identified pocket as a binding site for therapeutic ligands, we performed molecular docking simulations using a library of 1017 FDA-approved drugs from the PubChem database. Figure 6 illustrates the binding of lumacaftor, a drug currently approved for cystic fibrosis treatment, to the surface pocket induced by the Val149Gly mutation in human *SOD1*. PubChem IDs of the tested ligands and their corresponding binding energies are summarized in Table S2. Notably, Val149 plays a critical role in the formation of the functional *SOD1* homodimer. Its substitution with glycine is thought to disrupt the dimerization process, thereby contributing to the pathogenesis of amyotrophic lateral sclerosis (ALS) [33]. Consequently, the identification of ligands capable of restoring or stabilizing dimerization may offer a promising therapeutic strategy for the treatment of ALS.

The absence of new surface pockets in all other MD simulations aligns with the concept of cryptic pockets, as recently proposed [34]. Without specific ligands, proteins may hide their binding sites, exposing them only under favorable conditions. However, standard MD simulations, such as those employed in this study, may not capture the slow internal motions required for cryptic pocket openings, highlighting the need to broaden our focus beyond transient surface pockets [35]. We recognize that exploiting these cavities for effective drug design is more complex than simply identifying them. Moreover, the mutated protein may be essential, or it may be non-essential but toxic when mutated, requiring different therapeutic approaches. The emerging AlphaFold-based procedures, which have successfully identified cryptic openings in protein structures [36], offer additional methodologies for monitoring and targeting novel pockets for MRD therapeutic development. Nevertheless, our structural analysis provides a powerful shortcut for prioritizing which MRD cases merit further experimental drug-design efforts. To facilitate these investigations, we have developed Orpha.net.ta Web (Available online: https://github.com/PieroNiccolai/orpha.net.ta, accessed on 17 September 2024), a tool for rapid structural analysis of MRD mutations listed in the Orpha.net database. For each MRD, Orpha.net.ta Web indicates whether mutations exist that deserve consideration for medicinal chemistry studies, correlating specific diseases with the corresponding clusters from our analysis.

## 4. Materials and Methods

### 4.1. Development of the Missense Variant Dataset

ClinVar (National Center for Biotechnology Information, Bethesda, MD, USA) [37] and Orpha.net (INSERM, Paris, France) served as the starting point for our analysis, providing essential data for the identification and classification of MRD-related missense variants. Orpha.net (available online: https://www.orpha.net/, accessed on 17 September 2024) is a comprehensive database that provides information on RDs and orphan drugs, with details on genetic conditions, associated phenotypes, and clinical guidelines. ClinVar (available online: https://www.ncbi.nlm.nih.gov/clinvar/, accessed on 17 September 2024) is a publicly accessible genomic database maintained by the National Center for Biotechnology Information (NCBI). It serves as an open repository for clinically relevant genetic variants, linking them to human phenotypes based on expert-reviewed and community-submitted reports.

All MRD-related genes were identified by their official HGNC gene symbols, and corresponding proteins were referenced using UniProt accession codes (e.g., P00441 for *SOD1*). Each mutation was documented using standard amino acid notation, indicating the wild-type residue, position, and mutant residue (e.g., Val149Gly). The complete dataset, including all identifiers and parameters used in our analysis, is provided in Table S1.

### 4.2. Structure Prediction and Analysis

All structural information was obtained using AlphaFold 2.3.1 (DeepMind, London, UK) [23] to complete the dataset for this structural analysis. AlphaFold predicts protein structures by analyzing amino acid sequences and estimating the distances between residue pairs, generating high-accuracy 3D models. For each residue, it provides a predicted local distance difference test (pLDDT) score, which indicates the reliability of specific regions within the structure. In this study, we included only predicted models with very high reliability, defined by an average protein pLDDT score > 0.8 and a pLDDT score > 0.8 at the specific residue position.

### 4.3. Solvent Accessibility Profiling and Burial Analysis

For the atomic depth calculations, we used the SADIC v. 2.0 [38], a tool specifically designed to analyze protein atomic depths. We provide the files generated by AlphaFold as input data for SADIC. The output consists of modified files that include an additional column reporting atomic depth values for each atom. SADIC-modified files were then used for topological analysis of MRD mutation sites and molecular graphic presentations.

### 4.4. Molecular Dynamics Simulations

To assess the actual possibility for pocket formation in proteins with surface mutations identified by SADIC, 250 ns molecular dynamics (MD) simulations in explicit water were performed to compare wild-type and mutant proteins listed in Table 2. Each simulation was carried out for 250 ns using a standardized multistep protocol.

Wild-type protein structures were obtained from the RCSB Protein Data Bank (PDB) (Research Collaboratory for Structural Bioinformatics, San Diego, CA, USA) [39], and mutant models were generated using the DUET tool [40], which predicts the structural impact of point mutations on protein stability. Prior to simulation, all structures were refined using PyMOD 3.0 (Department of Biochemical Sciences, Sapienza University, Rome, Italy) [41] with MODELLER 10.5 to reconstruct missing side chains and resolve any steric clashes.

System setup was carried out using the CHARMM-GUI platform (Lehigh University, Bethlehem, PA, USA) [42], with all molecular parameters assigned according to the CHARMM36m (March 2019) force field. Each protein was placed in a periodic triclinic box filled with explicit TIP3P water molecules. The box dimensions were adjusted according to the size of each protein, ensuring a minimum distance of 10 Å between any protein atom and the edge of the box. The system was neutralized with Na^+^ or Cl^−^ counterions.

Energy minimization was performed using the steepest descent algorithm for up to 5000 steps or until the maximum force fell below 100 kJ/mol/nm, to remove steric clashes and allow for initial relaxation of the solvent environment.

The equilibration phase consisted of two steps with position restraints applied to heavy atoms of the protein. First, a 100 ps NVT equilibration was performed using a V-rescale thermostat to stabilize the system temperature at 300 K. This was followed by a 100 ps NPT equilibration using a Nosé–Hoover barostat to maintain the pressure at 1 atm, with a damping factor of 1 ps^−1^.

Following equilibration, 250 ns MD simulations were conducted using GROMACS 2019.3 (Department of Biophysical Chemistry, Groningen University, Netherlands) [43] with GPU acceleration. A 2 fs integration time step was used, and all bond lengths involving hydrogen atoms were constrained using the LINCS algorithm. Trajectory frames were recorded every 10 ps for subsequent analysis. Simulation trajectories were analyzed using built-in GROMACS tools and custom Python 3.11.10 scripts. Structural stability was assessed by calculating the RMSD and RMSF of backbone atoms.

### 4.5. Molecular Docking Simulations

A molecular docking simulation was performed to assess the potential applicability of the identified pocket as a binding site for potential ligands. The protein structure was prepared as a receptor and converted to pdbqt format using OpenBabel v.3.1.0 [44], as implemented in the AutoDockFR suite [45]. A library of 1017 FDA-approved drug ligands was obtained from the PubChem database [46], converted into their three-dimensional coordinates in pdbqt format, and used as input for the docking procedure. A simulation box with dimensions of 14 Å × 10 Å × 14 Å was defined around the target site, centered at coordinates X: 26.50, Y: 15.00, Z: 31.70. Docking calculations were performed using AutoDock Vina v.1.1.2 (Molecular Graphics Lab, Scripps Research Institute, La Jolla, CA, USA) [47,48], with the following parameters: exhaustiveness = 8, number of modes = 10, and energy range = 3 kcal/mol. The default Vina scoring function was used without modifications. The receptor was treated as rigid, and no flexible side chains or constraints were applied.

## 5. Conclusions

In the AlphaFold era, the vast amount of available data for protein structures enables bioinformatics-driven approaches to provide valuable insights into developing drug-based strategies for treating MRDs. The use of Alphafold models in the proposed topological analysis of mutation sites assumes a monomeric view of protein structures, independent of their functional oligomeric assemblies. By systematically mapping MRD-related mutation sites, we have analyzed side-chain orientation and amino acid replacement patterns to identify structural alterations that may lead to the formation of druggable pockets, potentially allowing for ligand binding aimed at restoring normal protein function. Our findings suggest that MRD-associated mutations frequently introduce void spaces in protein surfaces, which could potentially host therapeutic ligands. We employed molecular dynamics simulations to evaluate the stability of these mutation-induced surface pockets for potential drug binding. While only one case in our limited simulation set revealed a druggable pocket, this finding points to the likely prevalence of cryptic pockets that may only become apparent under specific conditions or with more extensive sampling. This underscores both the challenge and opportunity in targeting such sites. By combining structural bioinformatics with computational modeling, our study underscores the potential of targeting mutation-induced structural changes in MRD proteins. Future research should explore more advanced simulation methods and experimental validation to assess the feasibility of ligand-based therapeutic interventions, considering the specific biological context of each mutation.

## Figures and Tables

**Table 1:** Cluster (#) distribution of mutated amino acids according to side chain orientation.

#	Side Chain Orientation	Amino Acids	Occurrences
1	*D_i_*_α_ ≤ 0.2 and D_iSC_ < D_iα_	Ala, Cys, Gly, Ile, Leu, Met, Phe, Val	1018
2	*D_iSC_ >* 0.5 and D_iSC_ > D_iα_	Tyr, Phe, Leu, Ile, Val, Trp, Met Asp, Glu, His, Lys, Arg	722
3	All other cases	all amino acids	3988

**Table 2:** Structural features of MRD-associated mutations for MD simulations.

Gene ^a^	PDB ^b^	Aaa *N* Bbb ^c^	D_iSC_/D_iα_ ^d^	*V/v* ^e^
SOD1	8GSQ	Val149Gly	1.46	2.37
SOD1	8GSQ	Leu127Ser	1.43	1.90
SOD1	8GSQ	Ile114Thr	1.23	1.45
SOD1	8GSQ	Ile152Thr	1.30	1.45
QDPR	1HDR	Trp108Gly	3.77	3.89
HOGA1	3S5N	Trp262Gly	1.37	3.89
TPK1	3S4Y	Phe132Ser	1.54	2.17
PAFAH1B1	7MT1	Phe142Ser	1.24	2.17

^a^ MRD-responsible gene; ^b^ ID structure from the PDB; ^c^ wild-type residue Aaa at position N replaced by residue Bbb; ^d^ the extent of outward side chain orientation; ^e^ the ratio between Aaa volume, V, and Bbb volume, where v is calculated according to [32].

## Data Availability

The data presented in this study are available on request from the corresponding author.

## Supplementary Materials

The following supporting information can be downloaded at https://www.mdpi.com/article/10.3390/ijms26094072/s1.
