# Supplementary material for: Profiling of Protein-Coding Missense Mutations in Mendelian Rare Diseases: Clues from Structural Bioinformatics
Source: Int J Mol Sci. 2025 Apr 25;26(9):4072. doi: 10.3390/ijms26094072 (PMC12071383; doi:10.3390/ijms26094072)
Supplement: Supplementary file 1 [file ijms-26-04072-s001.zip › SI.pdf]

International Journal of *Molecular Sciences*

MDPI

Supporting Information for

Structural Bioinformatics Profiling of Mendelian Rare Disease Mutations: Insights for Drug Design and Therapeutic Targeting

Anna Visibelli[1,+], Rebecca Finetti[1,+], Piero Niccolai[2], Annalisa Santucci[1,3*], Ottavia Spiga[1,3] and Neri Niccolai[1,2]

[1] Department of Biotechnology, Chemistry and Pharmacy, University of Siena, 53100 Siena, Italy
[2] Le Ricerche del BarLume Free Association, Ville di Corsano, Monteroni d'Arbia, 53014 Siena, Italy
[3] Industry 4.0 Competence Center ARTES 4.0 Viale Rinaldo Piaggio, 56025 Pontedera, Pisa, Italy

*Correspondence: annalisa.santucci@unisi.it
[+]Equally contribution

- Table S1. See the attached .pdf file "Table S1.xlsx"
- Tabel S2. See the attached .pdf file "Table S2.xlsx"

Legend for Table S1

Copyright: © 2025 by the authors. Licensee MDPI, Basel, Switzerland. This article is an open access article distributed under the terms and conditions of the Creative Commons Attribution (CC BY) license (https://creativecommons.org/licenses/by/4.0/).

**Columns Name**	**Description**
Gene	Name of the gene involved in the mutation
Conditions	Condition or disease associated with the mutation
UniProt	Uniprot code of the corresponding protein
WT_Residue	Wild type amino acid residue in the protein sequence
Position	Residue position in the protein sequence
Mut_Residue	Aminoacid residue in which the original is mutated
DiSC	Depth index calculated for the side chain of the original amino acid, indicating the depth of the residue's side chain within the structure.
Diα	Depth index calculated for the α carbon of the original amino acid, indicating the depth of the α carbon of the residue within the structure.

DiSC/Diα	Ratio of the depth index of the side chain (Sadic) to that of the α carbon (CA), indicating the relative exposure of the residue in the protein structure.
Cluster	- 1: Amino acid mutations with side chain pointing inwards (Ala, Cys, Gly, Ile, Leu, Met, Phe, Val). Parameters: Ca <= 0.2 and Sadic < CA.<br>- 2: Mutations of amino acids with bulky side chains pointing outwards (Tyr, Phe, Leu, Ile, Val, Trp, Met) and mutations of amino acids with charged side chains pointing outwards (Asp, Glu, His, Lys, Arg). Parameters: Sadic> 0.5 and Sadic> CA.<br>- 3: Does not fall into any of the above cases.
V	Volume of the original amino acid in the protein structure
v	Volume of the mutated amino acid in the protein structure.
V/v	Ratio of the volume of the original amino acid (V) to that of the mutated amino acid (v), to assess the structural impact of the mutation.

Legend for Table S2

Columns Name	Description
PubChem ID	Unique identifier assigned to each tested ligand in the PubChem database.
Binding Energy	Calculated binding energy from molecular docking simulations, indicating the ligand's binding affinity to the target pocket.

Disclaimer/Publisher's Note: The statements, opinions and data contained in all publications are solely those of the individual author(s) and contributor(s) and not of MDPI and/or the editor(s). MDPI and/or the editor(s) disclaim responsibility for any injury to people or property resulting from any ideas, methods, instructions or products referred to in the content.
